# A PILOT RANDOMIZED CLINICAL TRIAL TO PROMOTE POSITIVE AFFECT IN PATIENTS WITH FIBROMYALGIA

**DOI:** 10.1093/geroni/igac059.428

**Published:** 2022-12-20

**Authors:** Cary Reid, Anthony Ong, Judy Moskowitz, Mubarak Sanni, Patricia Kim, Elaine Wethington, Elizabeth Addington, Erica Sluys

**Affiliations:** Weill Cornell Medicine, New York City, New York, United States; Cornell University, Ithaca, New York, United States; Northwestern University, Chicago, Illinois, United States; Weill Cornell Medicine, New York City, New York, United States; Weill Cornell Medicine, New York City, New York, United States; Cornell, Ithaca, New York, United States; Northwestern University, Chicago, Illinois, United States; Weill Cornell Medicine, New York City, New York, United States

## Abstract

Fibromyalgia syndrome (FMS) is a chronic musculoskeletal condition characterized by widespread pain, impaired physical functioning, and deficits in positive affect (PA). Standard behavioral therapies for FMS focus on reducing negative affect (NA) and yield only modest benefits. We examined the feasibility, acceptability and preliminary efficacy of a 6-week, online, PA-enhancing intervention, entitled: Lessons in Affect Regulation to Keep Stress and Pain UndeR control (LARKSPUR). We enrolled participants from one large health system in New York City and ResearchMatch. Ninety-five FMS patients were randomized to LARKSPUR or a neutral emotion reporting arm. Results to date indicate that LARKSPUR is feasible: recruitment (82%), retention (93%); and acceptable: mean satisfaction score on 1-10 scale (9.4, SD=1.2). Planned efficacy analyses will evaluate treatment effects on pain intensity and interference, depressive symptoms, and stress appraisals. If effective, LARKSPUR will lay the foundation for understanding how behavioral treatments for FMS can be optimized through PA enhancement.

